# Size-Induced Highly Selective Synthesis of Organometallic Rectangular Macrocycles and Heterometallic Cage Based on Half-Sandwich Rhodium Building Block

**DOI:** 10.3390/molecules27123756

**Published:** 2022-06-10

**Authors:** Li-Long Dang, Tian Chen, Ting-Ting Zhang, Ting-Ting Li, Jun-Liang Song, Ke-Jia Zhang, Lu-Fang Ma

**Affiliations:** 1 Henan Province Function-Oriented Porous Materials Key Laboratory, College of Chemistry and Chemical Engineering, Luoyang Normal University, Luoyang 471934, China; ctian2022@163.com (T.C.); zhangtingting131@126.com (T.-T.Z.); tingtinglichem@163.com (T.-T.L.); jlsong2022@163.com (J.-L.S.); kejiazhang2022@163.com (K.-J.Z.); mazhuxp@126.com (L.-F.M.); 2 Shanghai Key Laboratory of Molecular Catalysis and Innovative Materials, Fudan University, Shanghai 200433, China; 3 College of Chemistry, Zhengzhou University, Zhengzhou 450001, China

**Keywords:** half-sandwich fragment, macrocycle, molecular cage, host–guest chemistry

## Abstract

The controlled synthesis of organometallic supramolecular macrocycles cages remains interesting and challenging work in the field of supramolecular chemistry. Here, two tetranuclear rectangular macrocycles and an octuclear cage were designed and synthesized utilizing a rigid and functionalized pillar linker, 2,6-bis(pyridin-4-yl)-1,7-dihydrobenzo [1,2-d:4,5-d′]diimidazole (BBI4PY) based on three half-sandwich rhodium building blocks bearing different sizes. X-ray crystallography in combination with ^1^H NMR spectroscopy elucidated that the two building blocks with shorter spacers only result in rectangular macrocycles. However, the building block of bulkier size to avoid the π-π stacking interactions between two ligands BBI4PY led to the formation of an octuclear cage complex. The latter cage contains two types of metal ions, namely Rh^3+^ and Cu^2+^, showing significant characteristics of heterogeneous metal-assembling compounds. In addition, the cage accommodates two free isopropyl ether solvent molecules, thus displaying host–guest behavior.

## 1. Introduction

In recent years, the design and synthesis of coordination supramolecular compounds have made tremendous advances due to their various potential applications in catalysis, host–guest chemistry, nanomaterials, separation, etc. [1,2,3,4,5,6,7,8]. These studies have attracted the attention of many scientists and have gradually become a hot topic in the field of supramolecular chemistry, as demonstrated by the work of Sauvage, Stoddart, Stang, Jin and others [9,10,11,12,13]. Accompanied by the development of this field, a series of molecular knots [14,15,16,17], catenanes [17,18,19], Borromean rings [20,21,22], Solomon links [23,24,25] and molecular cages [26,27,28] have been designed reasonably and reported continuously. In the process of research, the main synthetic methods were divided into two classes: metal-ion-templated and template free based on coordination-driven methods [29,30,31,32,33]. The former has been utilized to construct various coordination supramolecular compounds. The latter can not only achieve the construction of supramolecular structures but can also study the structural dynamic transformation characteristics among molecular catenanes, Borromean rings, Solomon links or knots and organometallic macrocycles [22]. These studies have promoted the rapid development of supramolecular chemistry to a certain extent. However, the research on the selective formation of macrocycle and cage induced merely by the size effect of building blocks has never been reported. Additionally, in order to realize the selective construction of organometallic macrocycle and cage, the judicious selection of metallic building blocks and organic ligands with functional sites is vitally important.

The half-sandwich rhodium metal fragments [Cp*M] (M = Ir, Rh; Cp* = *η*^5^-pentamethyl-cyclopentadienyl) have always been used to construct various intriguing supramolecular structures as building blocks, such as supramolecular cages, knots, catenanes, Borromean rings and a Solomon link because of their directional coordination, good solubility and crystallization [34,35,36,37].

**Scheme 1:**
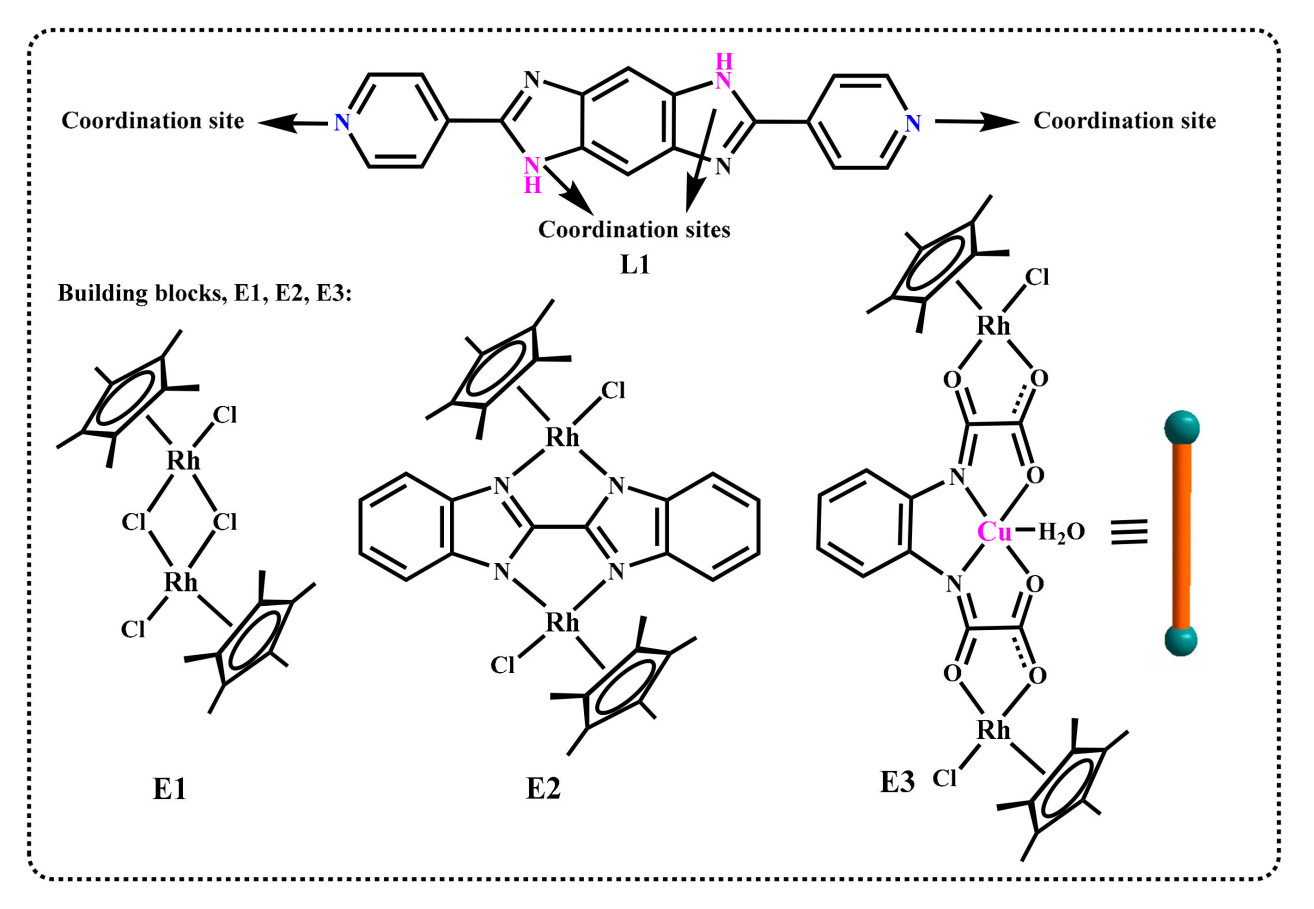
The designed ligand **L1** and building blocks **E1**, **E2** and **E3**.

Here, we designed and synthesized a new organic ligand, 2, 6-bis (pyridin-4-yl)-1, 7-dihydrobenzo [1, 2-d: 4, 5-d′] diimidazole (BBI4PY). Meanwhile, three Cp* Rh-based building blocks (Cp* = *η*^5^ − C_5_Me_5_) bearing different sizes and traits were carefully selected (Scheme 1). Based on a coordination-driven self-assembly strategy, three supramolecular compounds were realized smoothly, including two organometallic macrocycles and a heterometallic cage. In addition, these complexes were determined by single-crystal X-ray diffraction (Supplementary Materials Table S1), elemental analysis (EA), infrared spectroscopy (IR), thermogravimetric analysis (TGA) and ESI-MS.

## 2. Results

### 2.1. Synthesis of Metallarectangles **1**, **2** Based on **E1** and **E2**

**Scheme 2:**
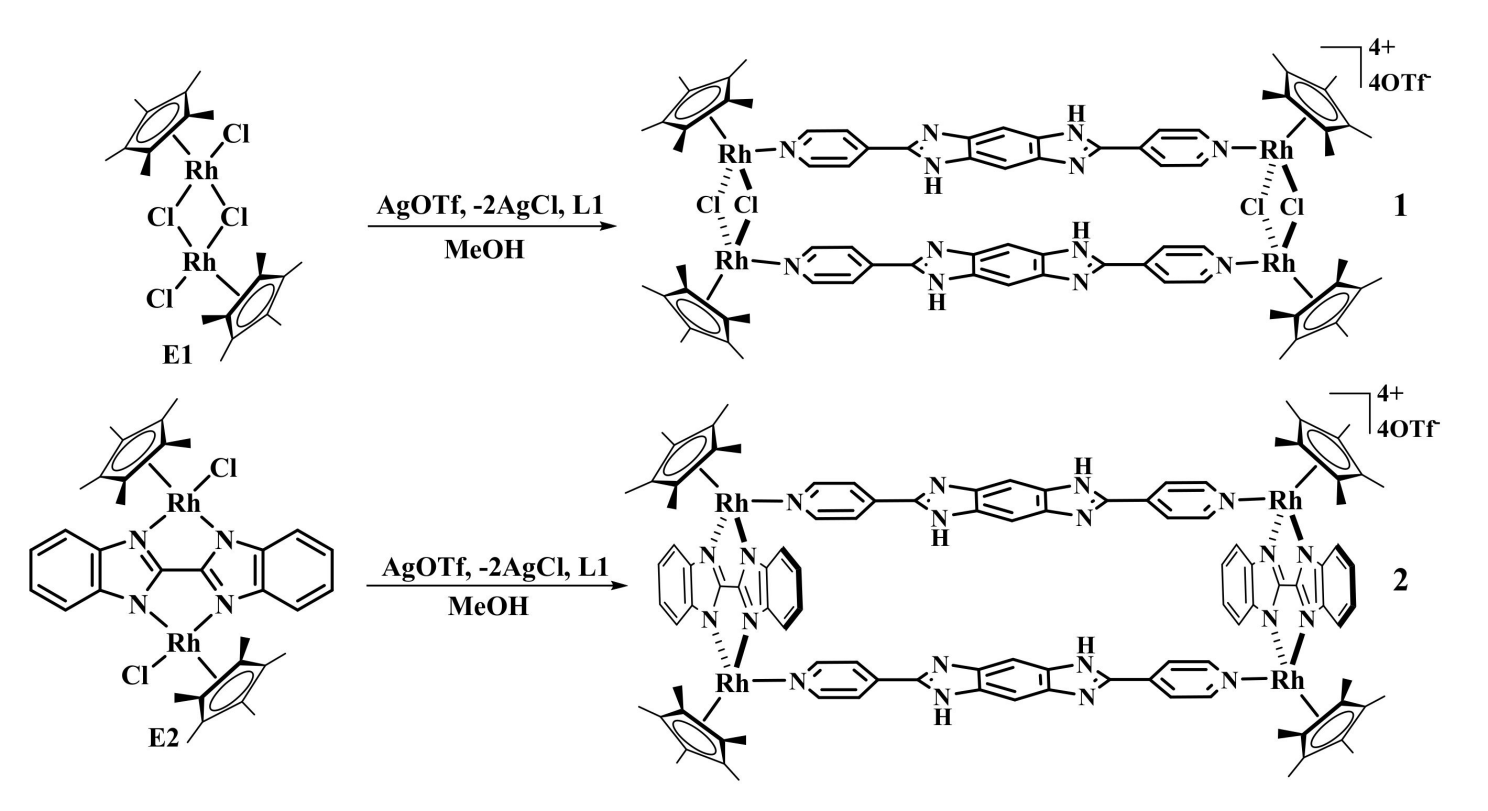
Synthesis of metallarectangle compounds (**1**, **2**).

The ligand **L1** and two binuclear complexes [Cp*RhCl(μ-Cl)]_2_ (**E1**) [38] [Cp*_2_Rh_2_(BiBzIm)]Cl_2_ (**E2**) [39] were prepared according to literature methods. The metallarectangles **1**, **2** were acquired by the combination of **L1** ligand and the binuclear complexes following a two-step construction method. As shown in Scheme 2, firstly, the binuclear complex was treated with two equivalents of silver trifluoromethanesulfonate (AgOTf) in the dark so that it could be connected to the bridging donor ligand. Followed by addition of the pyridine linker **L1** ligand, the crystalline products **1**, **2** were obtained after extraction with methanol/isopropyl ether in yields of 86% and 83%, respectively. Single crystals of sufficient quality for X-ray diffraction were obtained by slow diffusion of isopropyl ether into solutions of both complexes in methanol at ambient temperature over three days.

### 2.2. Analysis of Metallarectangle **1**

**Figure 1:**
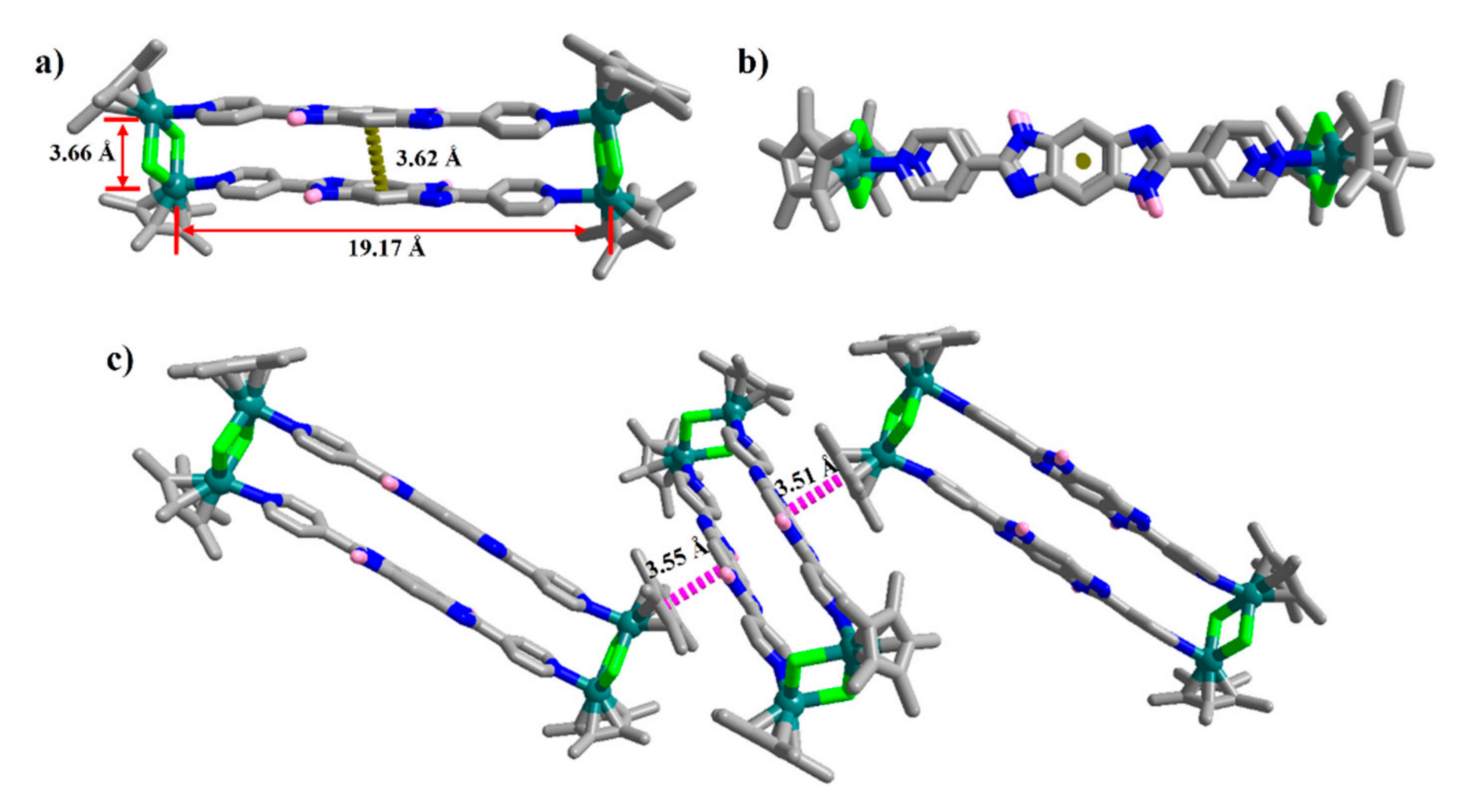
(**a**) Crystallographically determined molecular structure of the main cation of complex **1** and the π−π accumulation effect (3.62 Å) in **1**; (**b**) top view of **1**; (**c**) accumulation pattern of compound **1** and π−π intermolecular interaction (3.55 and 3.51 Å). All hydrogen atoms were omitted for clarity (N—blue; C—gray; Rh—Aqua; Cl—green).

Single-crystal X-ray structure determination and NMR spectroscopy confirmed the nature of complex **1**. As shown in Figure 1, the two binuclear (RhIII) molecular clips **E1** are connected by two rigid dipyridyl ligands **L1**, adopting a rectangular macrocyclic compound **1**. The structural analysis of this complex shows short and long Rh-Rh nonbonding distances of 3.66 and 19.17 Å, respectively. The separation between central conjugated planes from two ligand **L1** is about 3.62 Å, displaying strong π-π stacking interaction possibly due to the electron delocalization effect of benzimidazole units. In addition to the intramolecular π-π stacking, similar interactions could also be found between these discrete metallarectangle molecules, in which the separation between Cp* group of one metallarectangle molecule and phenyl unit from another metallarectangle molecule is about 3.51 of 3.55 Å, demonstrating that these discrete molecules coexist stably through a series of π-π stacking interactions.

**Figure 2:**
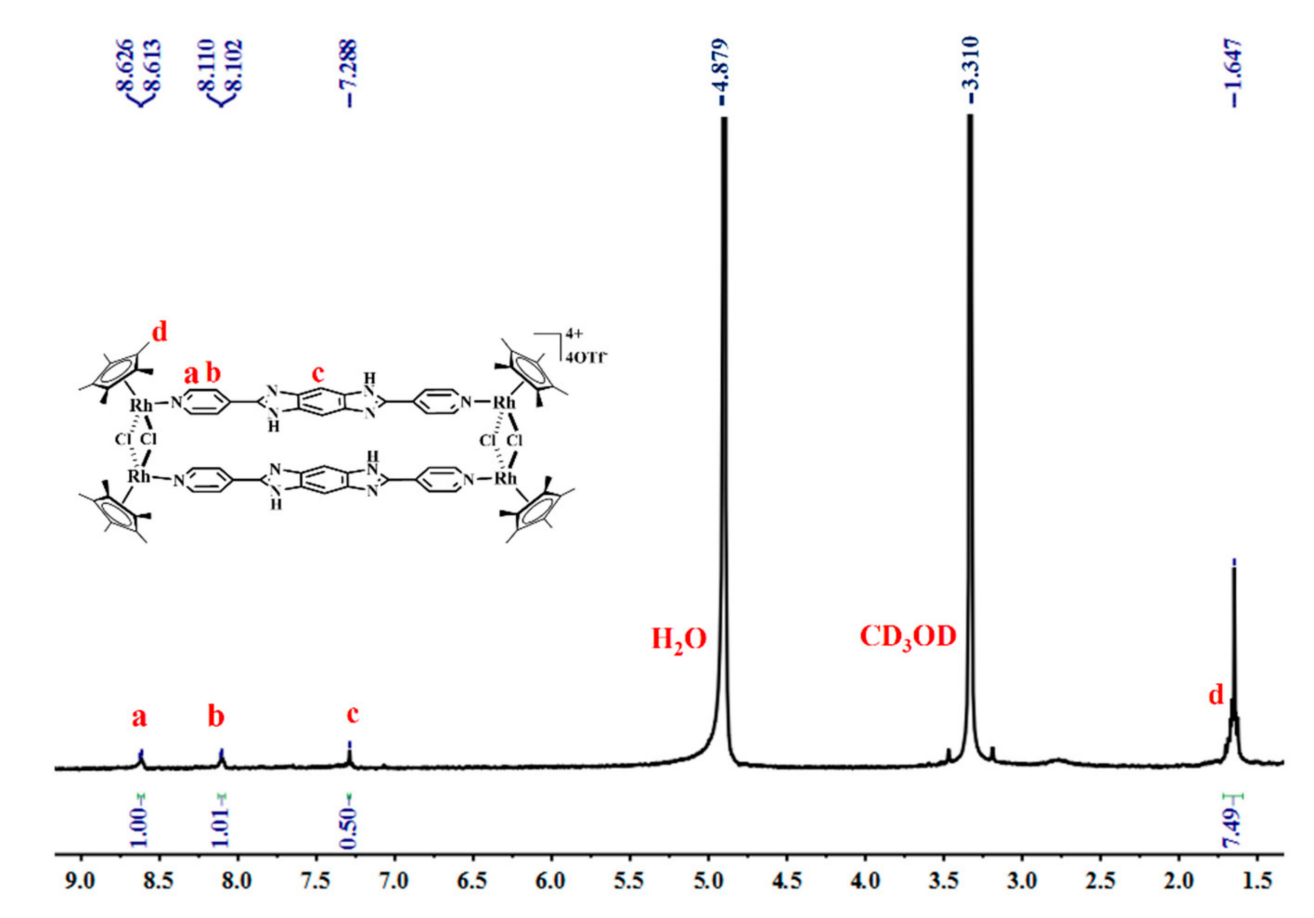
The ^1^H NMR (500 MHz, CD_3_OD, ppm) for **1** (6.0 mM, with respect to Cp*Rh).

**Figure 3:**
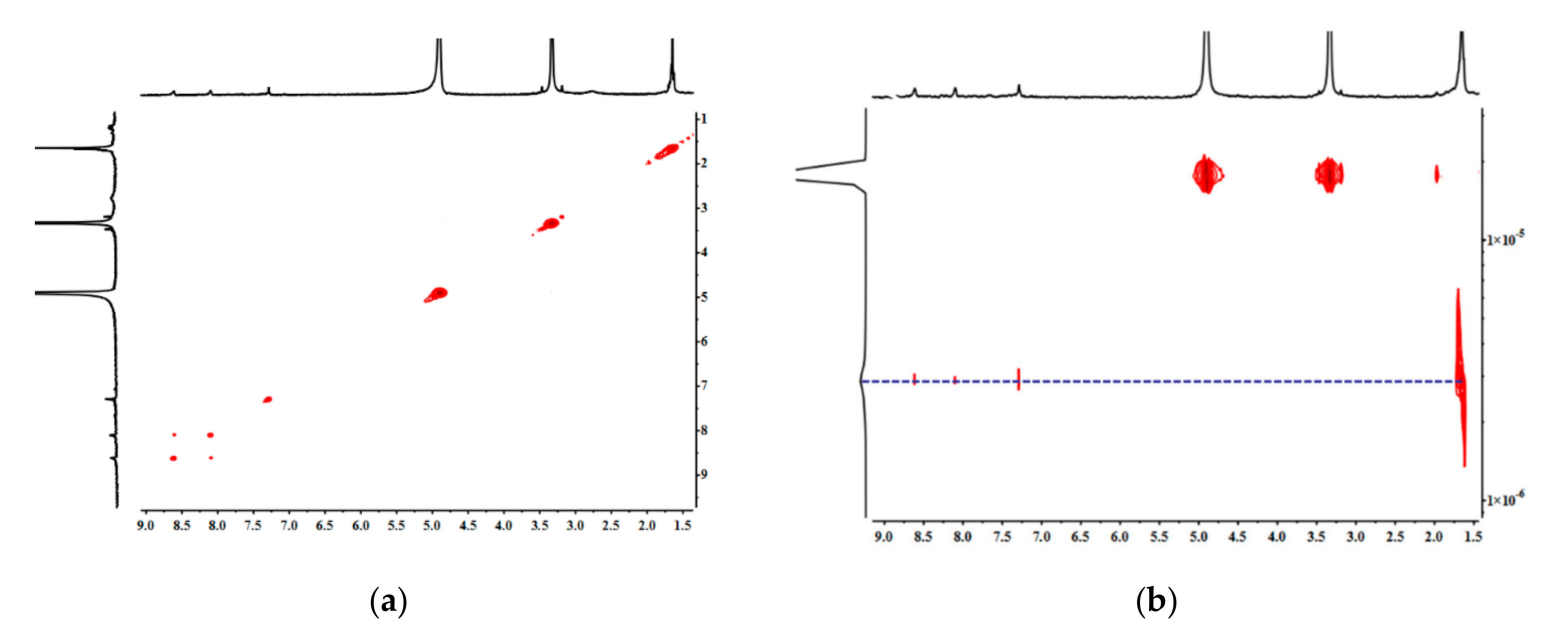
(**a**) The ^1^H−^1^H COSY NMR (500 MHz, CD_3_OD, ppm) for **1** and (**b**) the ^1^H−^1^H DOSY NMR (500 MHz, CD_3_OD, ppm) for **1** (2.38 × 10^−10^ m^2^s^−1^) (6.0 mM, with respect to Cp*Rh).

The presence of compound **1** in solution was also determined by the ^1^H NMR spectra (Figure 2), along with the combination of ^1^H−^1^H COSY NMR and ^1^H−^1^H DOSY NMR spectra (Figure 3a). In complex **1**, two obvious double peaks could be observed in 8.20 and 8.11 ppm, which could be attributed to two pyridine protons of ligand **L1**. Meanwhile, a strong singlet could be found in 7.29 ppm, belonging to phenyl protons of ligand **L1**. Moreover, the peak of the Cp* group proton was located at 1.65 ppm. The ^1^H diffusion-ordered spectroscopy (DOSY) NMR spectrum of **1** clearly indicated that these signals for the aromatic and Cp* units showed a single diffusion coefficient (2.38 × 10^−10^ m^2^s^−1^), further confirming stable presence of structure **1** (Figure 3b).

**Figure 4:**
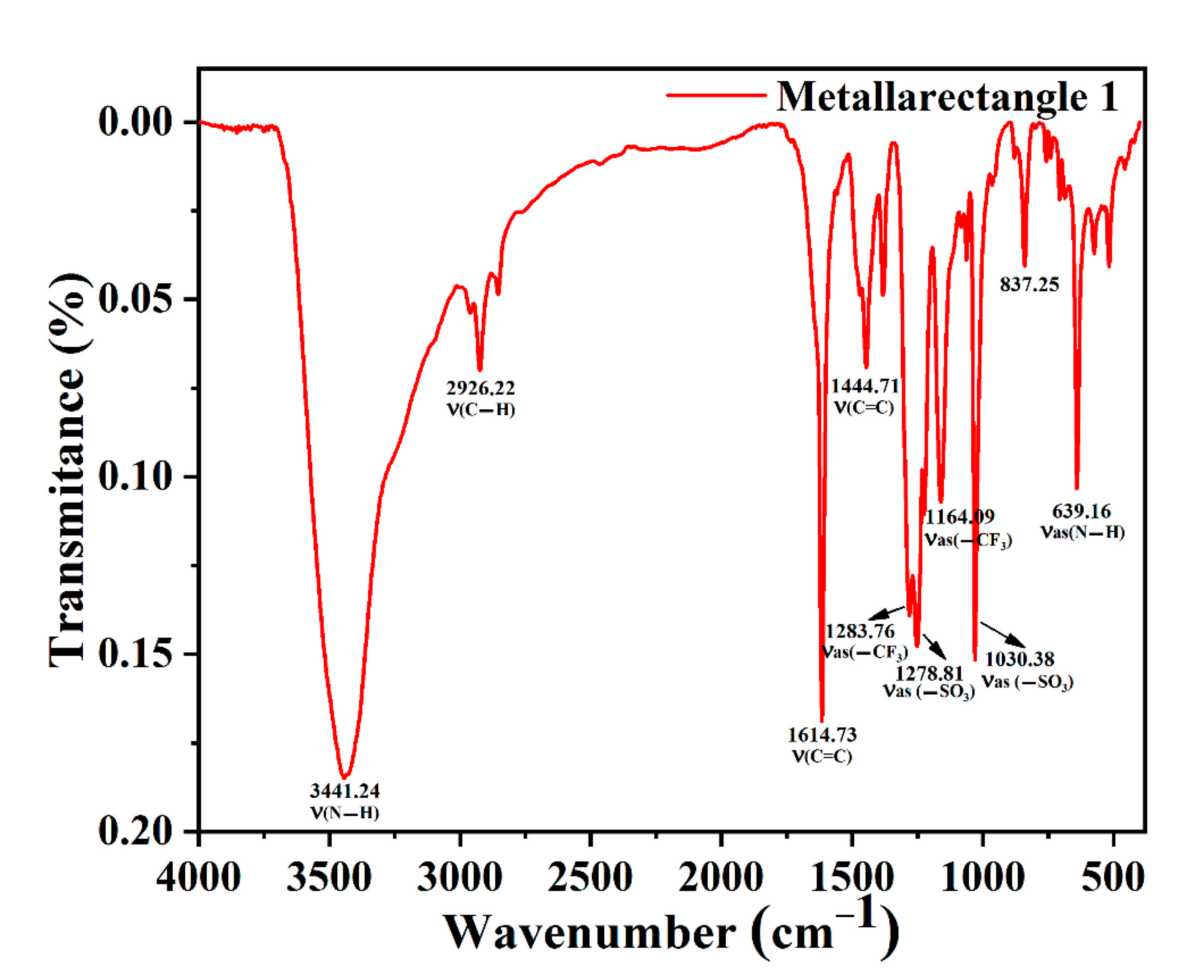
The IR spectrum of metallarectangle **1**.

Moreover, the results of IR spectrum of complex **1** exhibits a strong band at 3441 cm^−1^ owing to the stretching vibration of the N-H bond of imidazole group [40,41,42], the peaks at 1030 and 1279 are likely the asymmetric and symmetric sulfonate stretches and the peaks at 1160 are due to CF_3_ asymmetric and symmetric stretches [43,44,45]. The peaks at 1614 and 1444 are likely attributed to the vibration of aromatic ring structures of ligand **L1** (Figure 4).

### 2.3. Analysis of Metallarectangle **2**

**Figure 5:**
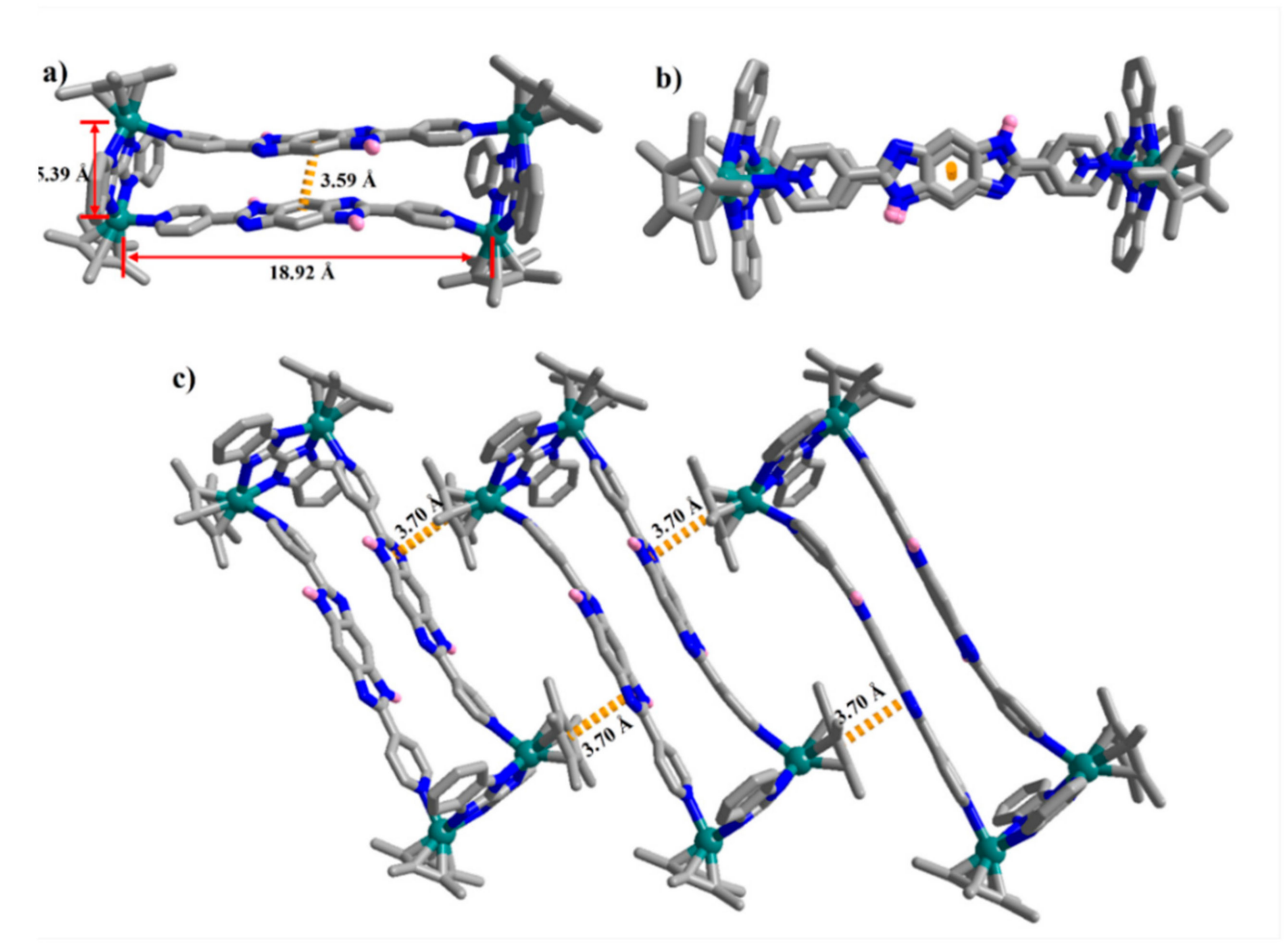
(**a**) Crystallographically determined molecular structure of the main cation of complex **2** and the π-π accumulation effect (3.59 Å) in **2**; (**b**) top view of **2**; (**c**) accumulation pattern of compound **2** and π–π intermolecular interaction (3.70 Å). All hydrogen atoms were omitted for clarity (N—blue; C—gray; Rh—aqua).

The solid-state structure of complex **2** was also demonstrated by single-crystal X-ray diffraction analysis, which shows a centrosymmetric rectangular macrocyclic compound with four rhodium atoms located at the corners. Here, the short Rh-Rh nonbonding distance is 5.39 Å, which is longer than that in **1** (3.66 Å) due to the larger spacer. The long Rh-Rh nonbonding distance of 18.92 Å is closely comparable to that measured in compound **1**. Interestingly, the distance of central conjugated units from two ligands **L1** in complex **2** is 3.59 Å, which is even shorter than that in complex **1**, reflecting that the central conjugated planes tend to be close to each other for forming π-π stacking interaction (Figure 5). Therefore, the imidazole units in **L1** are not coordinated with the rhodium atom, possibly due to the building blocks **E1**, **E2** providing a suitable distance for the central conjugated planes from two **L1** ligands, which is more conducive to forming strong π-π stacking interactions from electron delocalization effects of benzimidazole units. In addition, compared to that in compound **1**, the intermolecular force is different. Two sets of obvious π-π stacking interactions could be found between Cp* groups and benzimidazole units in complex **2,** accompanied by a distance of 3.70 Å. From Figure 5, we could find that each molecular rectangle **2** is able to form four π-π stacking interactions from another two rectangles, resulting in a stable solid structure. 

**Figure 6:**
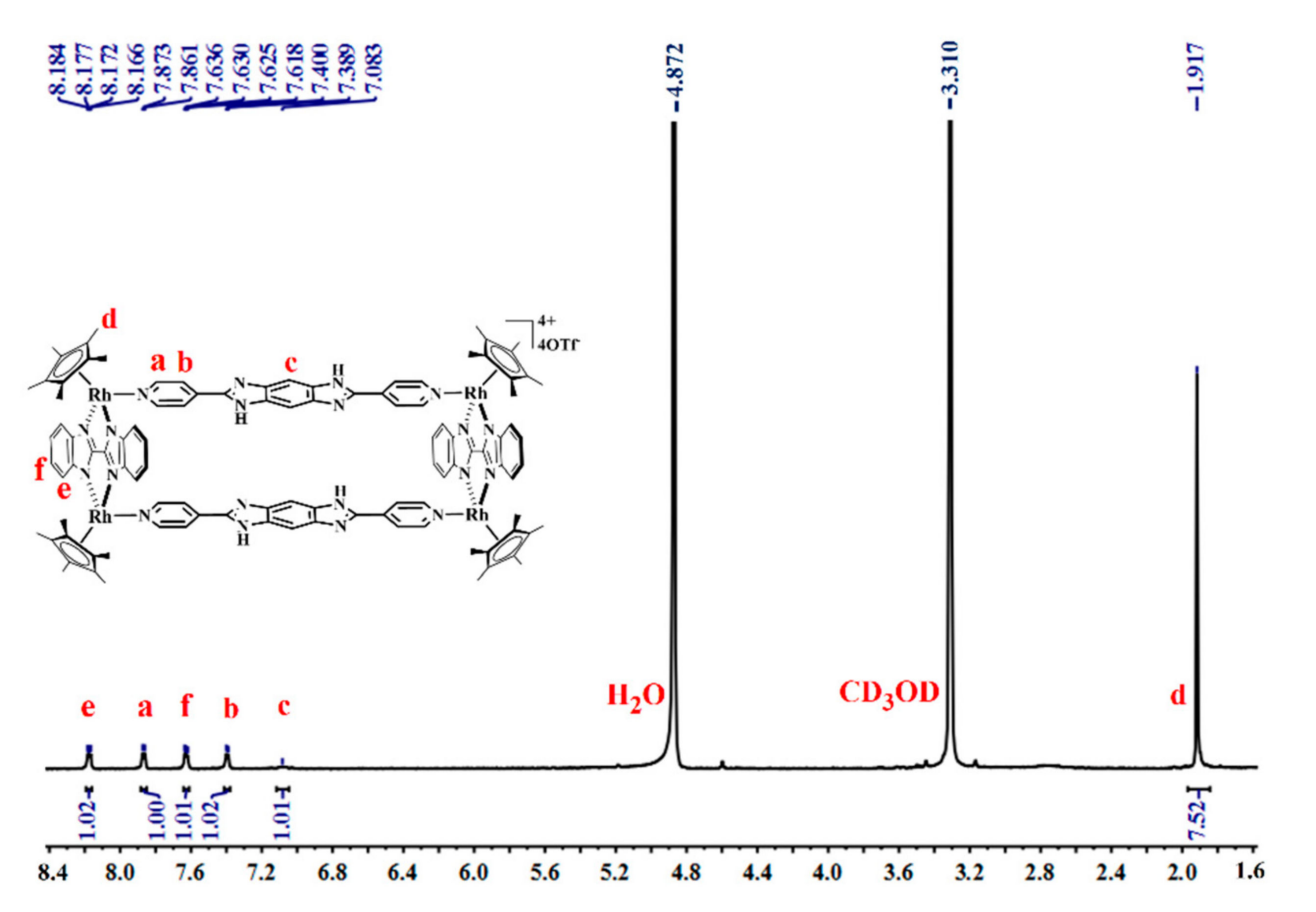
The ^1^H NMR (500 MHz, CD_3_OD, ppm) for **2** (12.0 mM, with respect to Cp*Rh).

**Figure 7:**
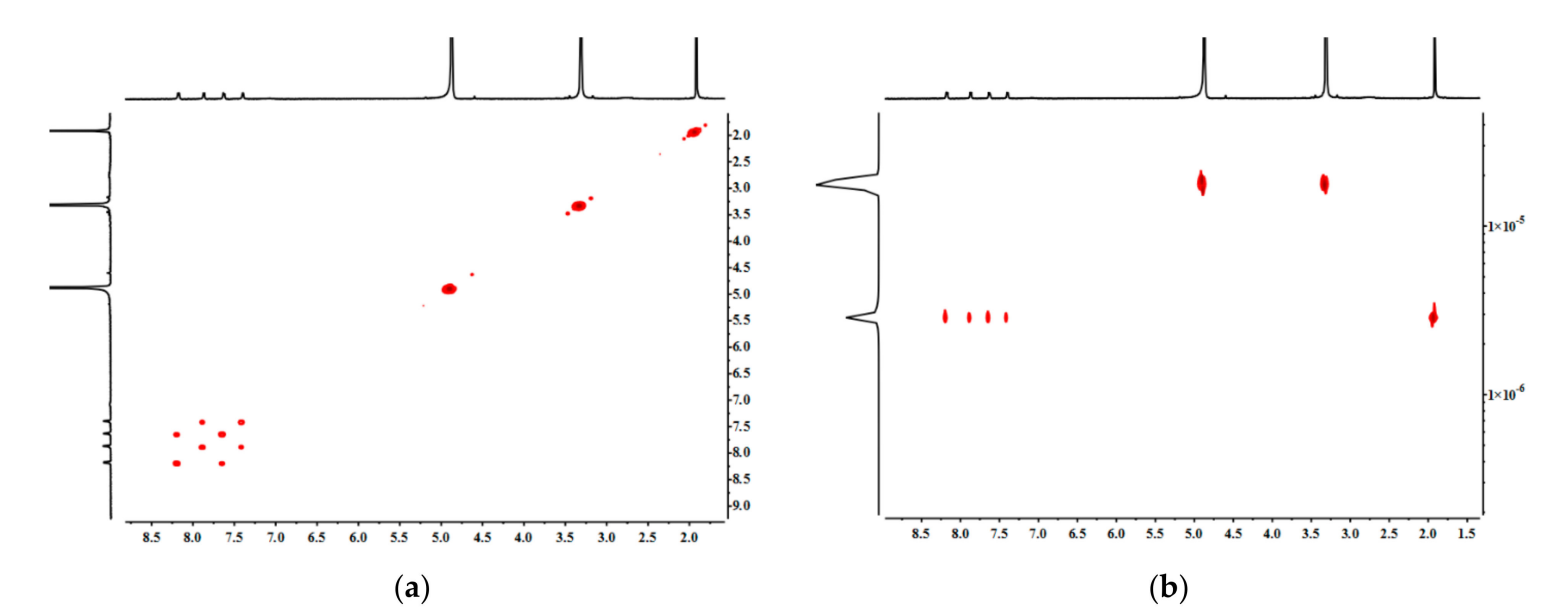
(**a**) The ^1^H−^1^H COSY NMR (500 MHz, CD_3_OD, ppm) for **2** and (**b**) the ^1^H−^1^H DOSY NMR (500 MHz, CD_3_OD, ppm) for **2** (2.45 × 10^−10^ m^2^s^−1^) (12.0 mM, with respect to Cp*Rh).

Moreover, the solution behavior of solid complex was also important for the various performance studies. Therefore, the solution characterization of complex **2** was also explored via the ^1^H NMR spectra accompanied by the two-dimensional spectra of ^1^H-^1^H COSY NMR and ^1^H-^1^H DOSY NMR (Figure 6 and Figure 7). Similarly, compared with that of complex **1**, two obvious multiple peaks 8.18–8.17, 7.64–7.62 ppm bearing clear coupling interaction can be observed in complex **2**, which belongs to the phenyl protons of **E2** building units. In addition, the signals of two pyridine and one phenyl proton of ligand **L1** could be found in 7.87, 7.39, 7.08 ppm, which is pretty close to that in complex **1**, albeit with a slight offset. The chemical shift of Cp* proton is at 1.92 ppm. Finally, a single diffusion coefficient from the ^1^H-^1^H DOSY NMR spectrum (2.45 × 10^−10^ m^2^s^−1^) clearly demonstrated the presence of complex **2** (Figure 7b).

**Figure 8:**
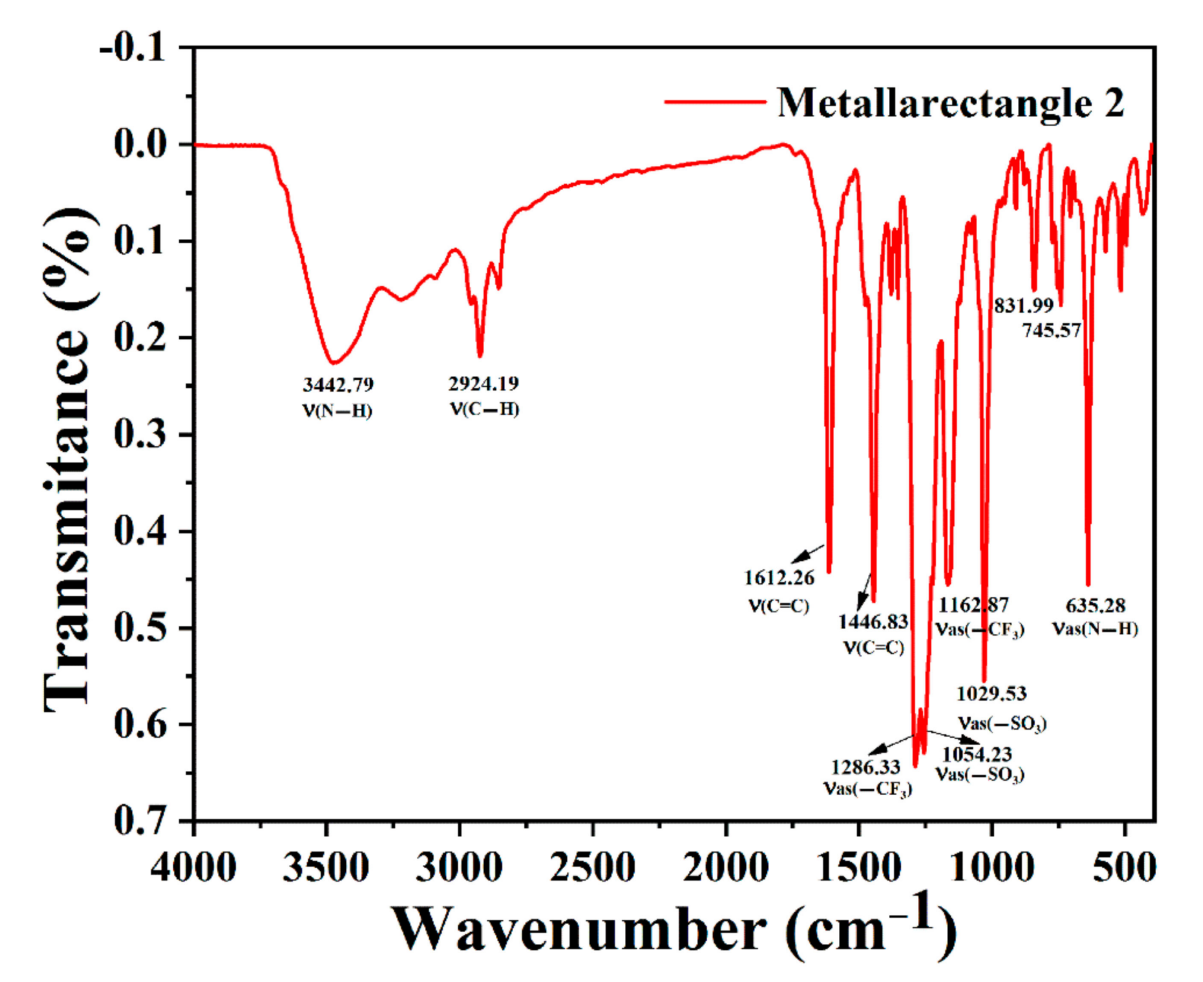
The IR spectrum of metallarectangle **2**.

Moreover, the IR spectrum of complex **2** was also carefully explored. Similar to complex **1**, the strong band at 3474 cm^−1^ could be observed owing to the stretching vibration of the N-H bond of imidazole group; the stretching vibrations of the OTf^−^ anions appeared at 1255, 1029, and 1163 cm^−1^, respectively. Additionally, the peaks at 1612 and 1445 are due to the vibration of aromatic ring of **L1**. [46,47,48,49,50]. In addition, a strong signal at 1474 cm^−1^ could be attributed to imidazole groups of building block **E2** (Figure 8).

### 2.4. Synthesis and Analysis of Heterometallic Cage **3@2iPr_2_O** Based on **E3**

**Scheme 3:**
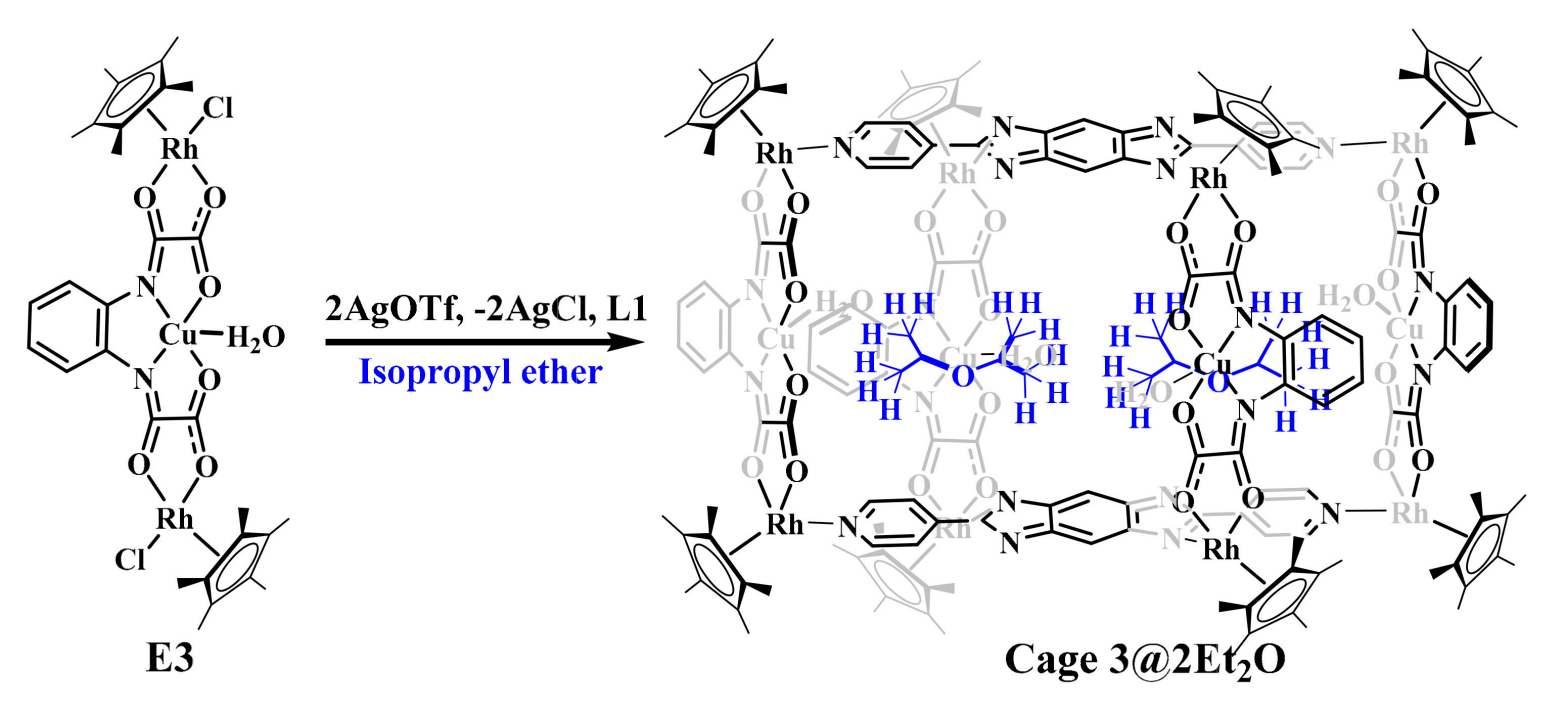
Synthesis of heterometallic cage **3@2iPr_2_O**.

Then, a longer heterometallic building block [Cp*_2_Rh_2_L^Cu^]Cl_2_ (L^Cu^ = [Cu(opba)]^2−^ [opba = o-phenylenebis(oxamato)] (**E3**) was chosen deliberately to build a larger assembly, where the Rh-Rh nonbonding distance is 10.10 Å, providing enough space to form additional coordination of two **E3** ligands through imidazole donors to Rh atoms. Through the self-assembly of ligand **L1** and building block **E3**, a new green centrosymmetric complex **3@2iPr_2_O** was obtained in a yield of 82% (Scheme 3). The structure was finally characterized by single-crystal X-ray diffraction, elemental analysis and UV-Vis spectra.

**Figure 9:**
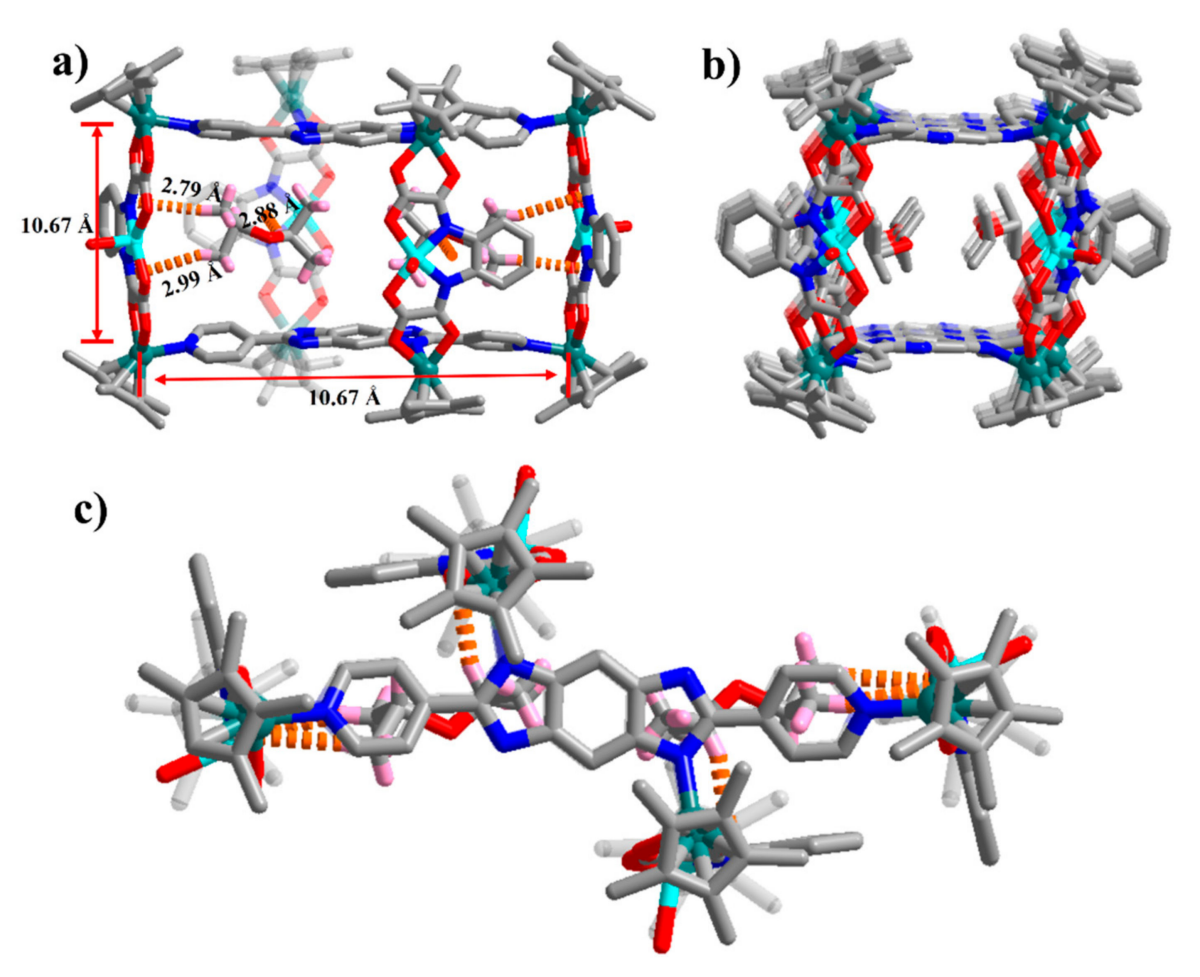
(**a**) Molecular structure of **3@2iPr_2_O**; (**b**) side view of **3@2iPr_2_O**; (**c**) top view of **3@2iPr_2_O**. Triflate anions and solvent molecules outside the framework, as well as hydrogen atoms, are omitted for clarity (N—blue; O—red; C—gray; Cu—cyan; Rh—aqua; H—pink).

Single crystals suitable for X-ray structure determination were obtained by slow vapor diffusion of isopropyl ether into a methanol solution of **3@2iPr_2_O** at room temperature, showing that a heterometallic cage was obtained in which two kinds of metal-positive ions, rhodium and copper ions, were included. The single-crystal structure displayed that the short Rh…Rh distance are ca. 8.67 Å from the two rhodium atoms coordinated to imidazole sites. In the building block **E3**, the Rh…Rh separation are ca. 10.67 Å. The longest Rh…Rh separation of 19.12 Å is similar to the distances found in complexes **1** and **2**. Interestingly, two isopropyl ether molecules are included in the host cage as two guest molecules by a series of C-H…π interactions, which was demonstrated by the thermogravimetric analysis (TGA). A careful structural analysis of complex **3@2iPr_2_O** displayed that each isopropyl ether molecule could form three C-H…π interactions with two building blocks **E3**. The distance between the H proton and the conjugated plane is about 2.79, 2.99 and 2.88 Å, and three interactions stabilize just one isopropyl ether molecules. Additionally, another isopropyl ether was stabilized by the other two building blocks **E3** by three C-H…π interactions. In addition, the shortest separation between the two isopropyl ether molecules is 3.03 Å. The distance avoids the interference of van der Waals forces between the two isopropyl ether molecules. Therefore, cage **3** can be used as a good host to selectively wrap the isopropyl ether molecules (Figure 9).

**Figure 10:**
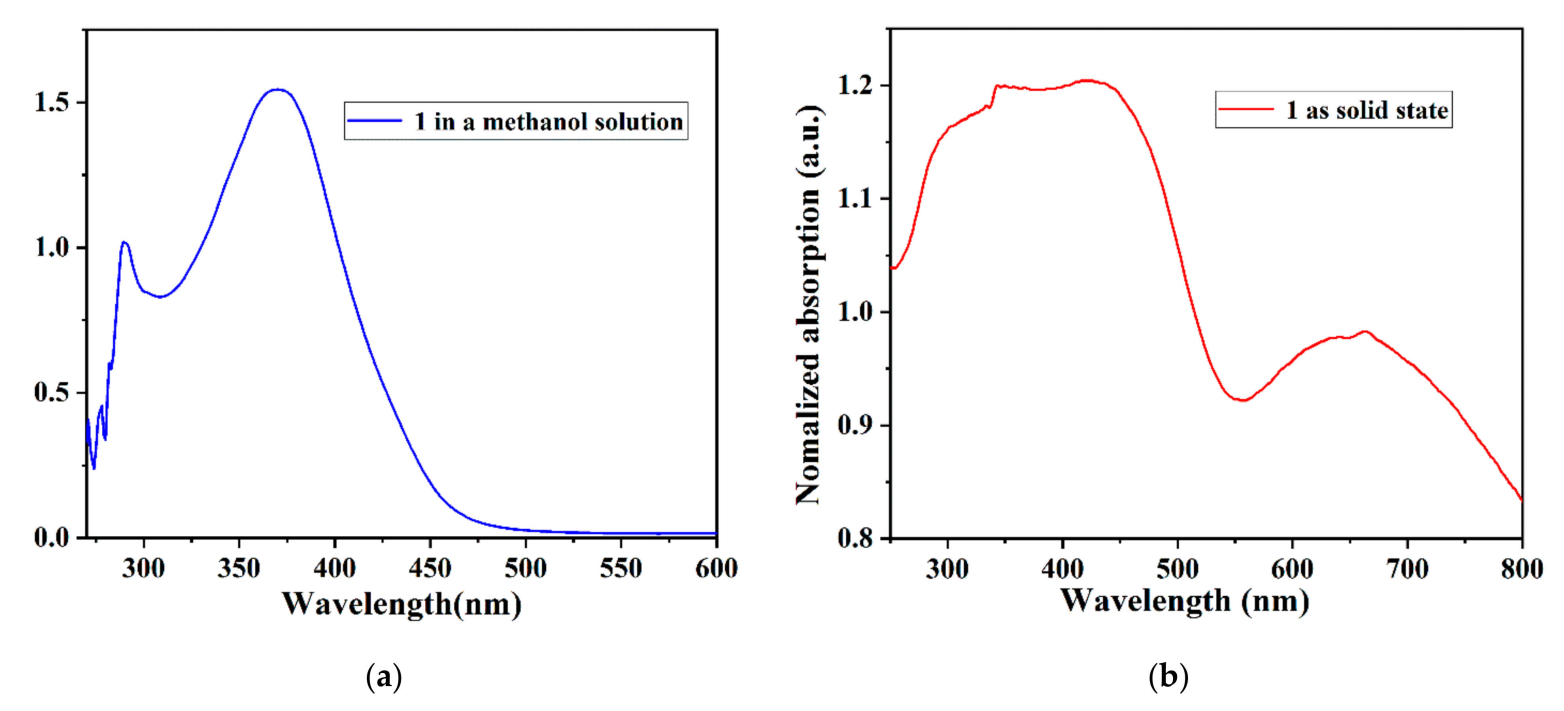
The liquid (**a**) and solid state (**b**) UV-vis spectroscopy exploration of cage **3@2iPr_2_O**.

**Figure 11:**
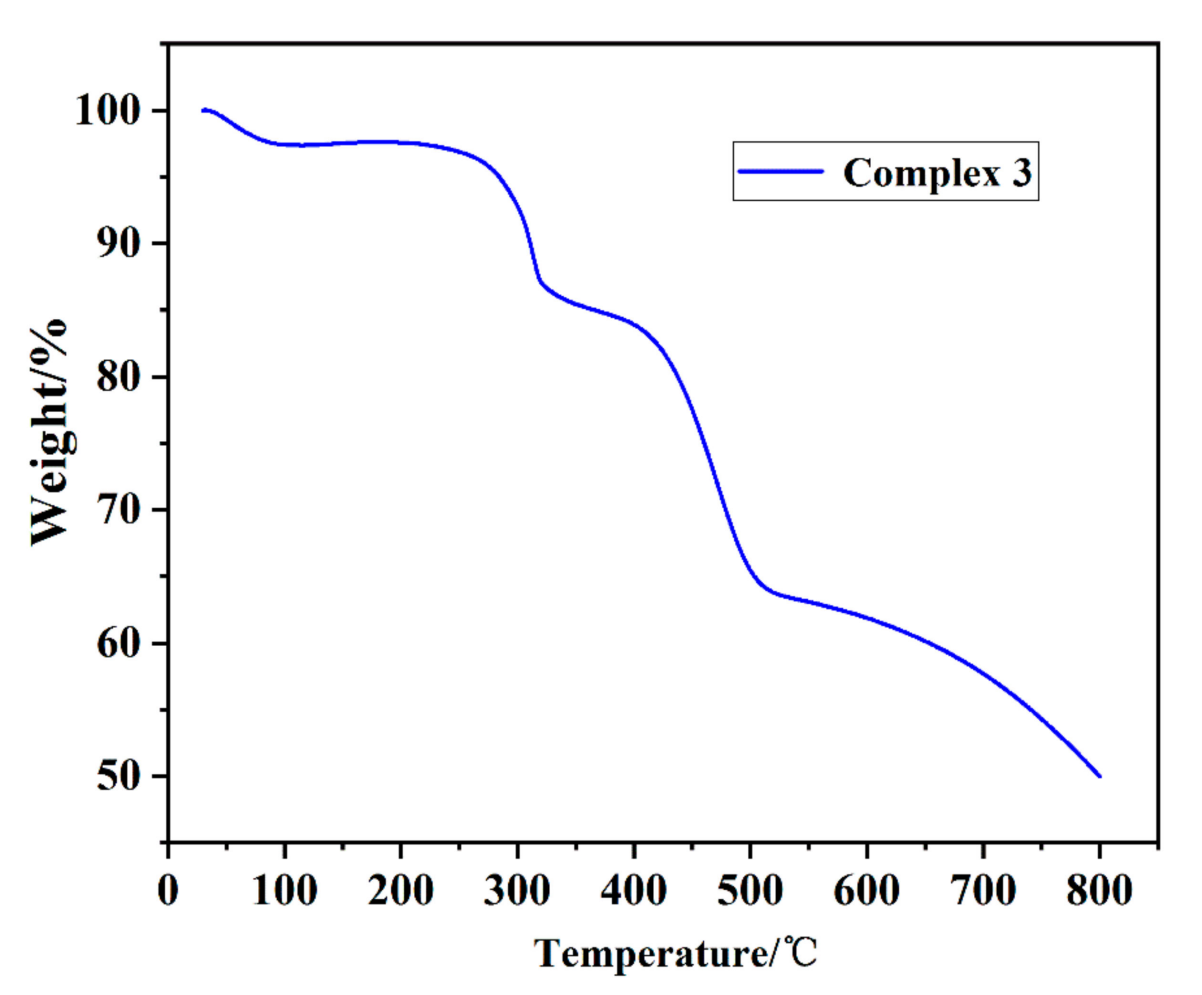
The TGA exploration of cage **3@2iPr_2_O**.

**Figure 12:**
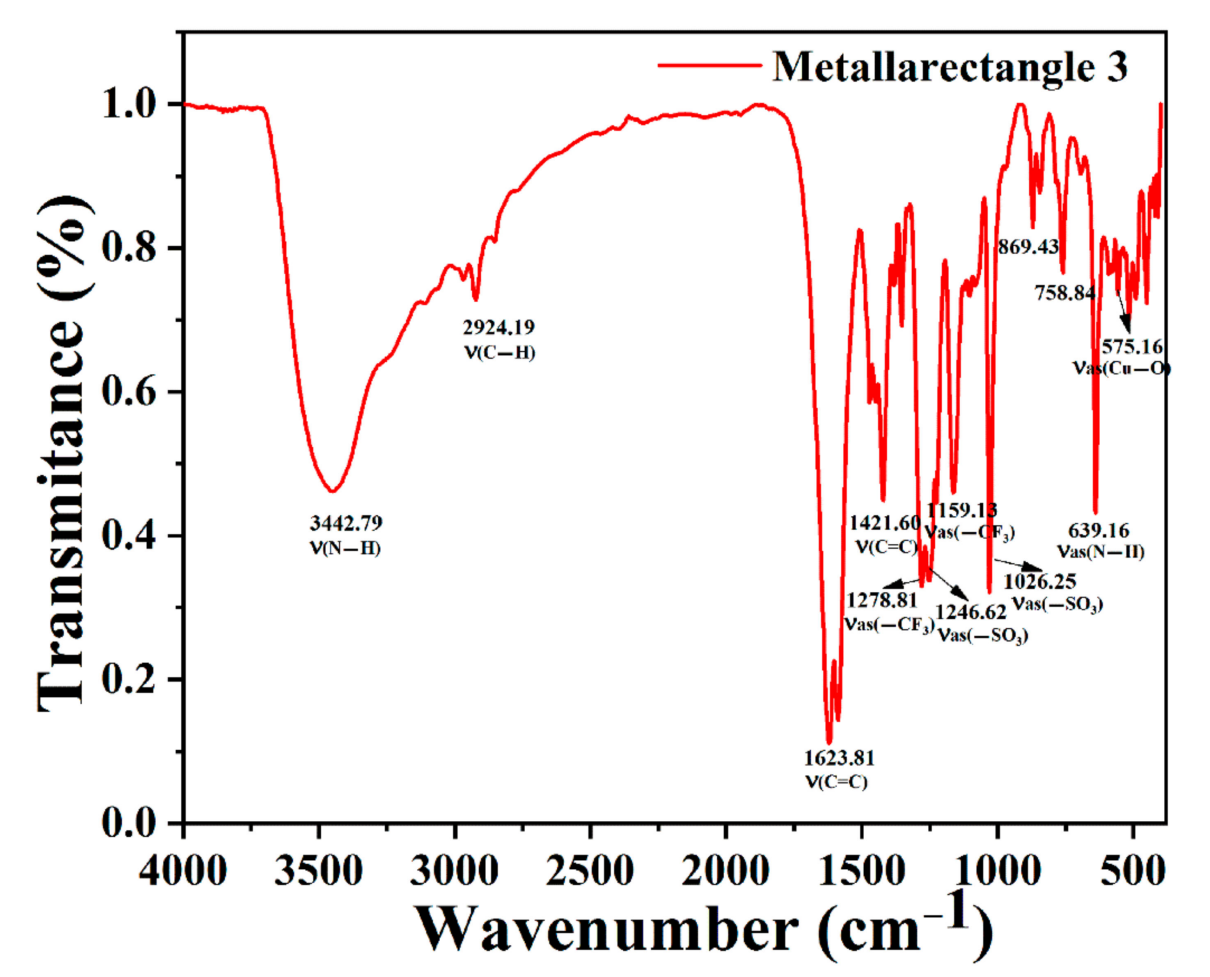
The IR spectrum of cage **3**.

Then, a series of tests, such as UV-vis (Figure 10), TGA and IR spectra were performed carefully to explore the structural characteristic of cage **3@2iPr_2_O**. The TGA test also showed that a significant loss was found in 30–100 °C, reflecting the loss of isopropyl ether and coordinated H_2_O molecules. Then, when the temperature rose from 100–300 °C, no mass loss was found, showing the structural stability. After that, an obvious weak loss (17.4%) can be observed, which is consistent with the mass of the two ligands **L1** (17.1%), suggesting the skeletal collapse of complex **3@2iPr_2_O** (Figure 11). The results also reflect a stronger interaction between the host framework and guest molecules contained in a cage than these free isopropyl ether molecules. The UV-vis in solution and solid state and IR spectrum tests both determined the structure of this complex **3@2iPr_2_O** (Figure 12). The structural analysis of IR spectrum for complex **3@2iPr_2_O** showed a strong band at 3442 cm^−1^, attributed to the stretching vibration of the N-H bond of imidazole group. Additionally, the stretching vibrations of the OTf^−^ anions appeared at 1278, 1026, and 1159 cm^−1^, respectively [51,52,53,54,55]. Additionally, the vibration of aromatic ring of ligand **L1** is likely at 1615 and 1421 cm^−1^ [56,57,58,59,60]. In addition, a strong signal at 575 cm^−1^ could be attributed to the Cu-O bonding signal of building block **E3**. (Figure 12).

### 2.5. The Host−Guest Chemistry Exploration of Heterometallic Cage **3@2iPr_2_O**

**Figure 13:**
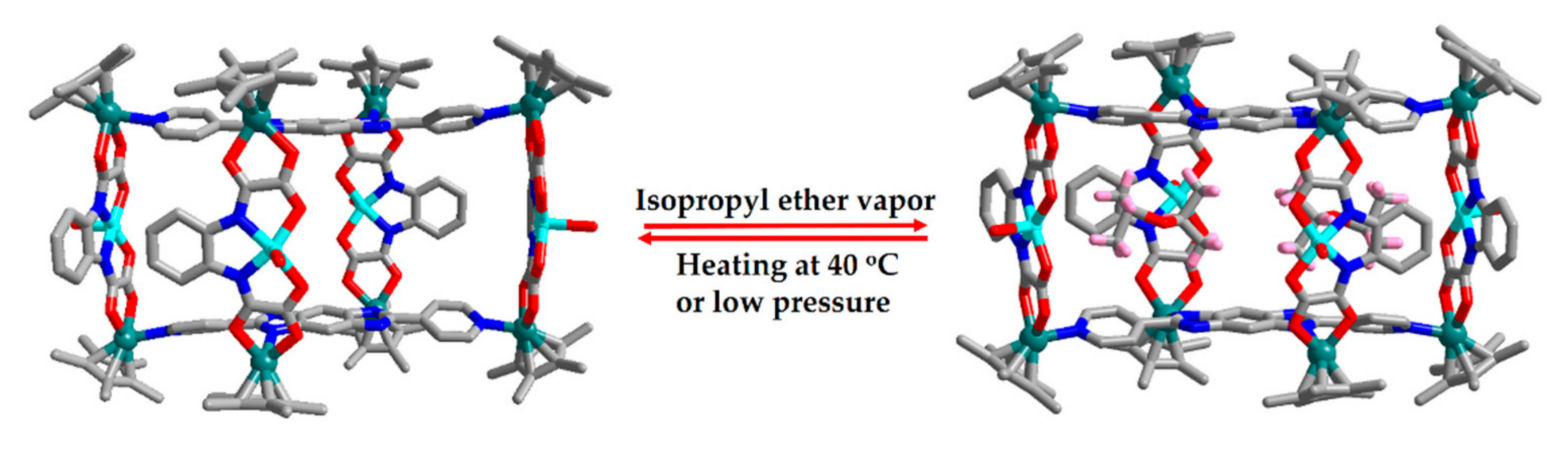
The capture and release process of cage **3** for isopropyl ether molecules.

In addition, when the crystals **3@2iPr_2_O** was allowed to vacuum under low pressure for 3h, after that, the isopropyl ether molecules were removed and the cage structure could be maintained integrally, which was demonstrated by the TGA tests (Figure 13). After that, the empty cage **3** was dissolved in a methanol solution. Then, the isopropyl ether solvents were allowed to diffuse into the above solution. After three days, good green crystals were recovered; the crystals could be determined by the single X-ray diffraction analysis, demonstrating the reformation of host–guest cage **3@2iPr_2_O** containing two isopropyl ether molecules. The results reflect that the cage **3@2iPr_2_O** packaging isopropyl ether molecules has cyclicity.

## 3. Experimental Section

### Materials

All reagents and solvents were obtained commercially and used without further purification. The starting materials [Cp*RhCl(μ-Cl)]_2_ (**E1**), [Cp*_2_Rh_2_(BiBzlm)](Cl)_2_ (**E2**) and [Cp*_2_Rh_2_(L^Cu^)]Cl_2_ (**E3**) were prepared according to literature methods. NMR spectra were recorded on Bruker AVANCE I 400 spectrometers at room temperature and referenced to the residual protonated solvent. Elemental analyses were performed on an Elementar Vario EL III analyzer. IR spectra of the solid samples (KBr tablets) in the range 400–4000 cm^−1^ were recorded on a Nicolet AVATAR-360IR spectrometer.

## 4. Conclusions

In summary, two metallarectangles were acquired successfully by a combination of ligand **L1** and two Cp*Rh-based building blocks **E1** and **E2** through a coordination-driven self-assembly strategy. Interestingly, through deliberately choosing a reasonable copper based building unit **E3**, a novel heterometallic cage was obtained smoothly, reflecting that size effect could affect the formation of different topological complexes. In addition, the cavity of the host cage contained two isopropyl ether molecules by multiple C-H…π interactions, showing an obvious host-chemistry effect. Additionally, these complexes were characterized by single-crystal X-ray diffraction analysis, NMR spectra and UV-Vis spectra. We believe that our results may be useful for controlling the synthesis of complicated structures and potential applications of organometallic macrocycles and a heterometallic cage, in addition to encouraging the design of further novel building blocks and functional architectures.

## Data Availability

Not applicable.

## Supplementary Materials

The following supporting information can be downloaded at: https://www.mdpi.com/article/10.3390/molecules27123756/s1, Materials and Methods Section, Table S1: Crystallographic data for 1, 2 and 3.

## Sample Availability

Samples of the compounds are available from the authors.

## References

[1] Percástegui E.G., Ronson T.K., Nitschke J.R. (2020). Design and applications of Water-Soluble coordination cages. Chem. Rev..

[2] Northrop B.H., Zheng Y.R., Chi K.W., Stang P.J. (2009). Self-Organization in Coordination-Driven Self-Assembly. Acc. Chem. Res..

[3] Clever G.H., Punt P. (2017). Cation–Anion arrangement patterns in Self-Assembled Pd_2_L_4_ and Pd_4_L_8_ coordination cages. Acc. Chem. Res..

[4] Ibáñez S., Poyatos M., Peris E. (2020). N-Heterocyclic carbenes: A door open to supramolecular organometallic chemistry. Acc. Chem. Res..

[5] Zhou Z., Wang Y., Peng F., Meng F., Zha J.J., Ma L., Du Y.H., Peng N., Ma L.F., Zhang Q.H. (2022). Intercalation-activated layered MoO_3_ nanobelts as biodegradable nanozymes for tumor-specific photo-enhanced catalytic therapy. Angew. Chem. Int. Ed..

[6] Zhao Y., Wang Y.J., Wang N., Zheng P., Fu H.R., Han M.L., Ma L.F., Wang L.Y. (2019). Tetraphenylethylene-Decorated Metal−Organic Frameworks as Energy-Transfer Platform for the Detection of Nitro-Antibiotics and White-Light Emission. Inorg. Chem..

[7] Yang X.G., Zhai Z.M., Lu X.M., Ma L.F., Yan D.P. (2020). Fast crystallization-deposition of orderly molecule level heterojunction thin films showing tunable up-conversion and ultrahigh photoelectric response. ACS Cent. Sci..

[8] Yang X.G., Qin J.H., Huang Y.D., Zhai Z.M., Ma L.F., Yan D.P. (2020). Highly enhanced UV-vis-NIR light harvesting and photoelectric conversion of pyrene MOF by encapsulation of D-π-A cyanine dye. J. Mater. Chem. C.

[9] Pöthig A., Casini A. (2019). Recent developments of supramolecular Metal-based structures for applications in cancer therapy and imaging. Theranostics.

[10] Shan W.L., Lin Y.J., Hahn F.E., Jin G.X. (2019). Highly selective synthesis of Iridium(III) metalla[2]catenanes through component Pre-Orientation by π⋅⋅⋅π stacking. Angew. Chem. Int. Ed..

[11] Chakrabarty R., Mukherjee P.S., Stang P.J. (2011). Supramolecular coordination: Self-Assembly of finite Two- and Three-Dimensional ensembles. Chem. Rev..

[12] Garci A., Beldjoudi Y., Kodaimati M.S., Hornick J.E., Nguyen M.T., Cetin M.M., Stern C.L., Roy I., Weiss E.A., Stoddart J.F. (2020). Mechanical-Bond-Induced exciplex fluorescence in an Anthracene-Based homo[2]catenane. J. Am. Chem. Soc..

[13] Denis M., Goldup S.M. (2018). A [3]Rotaxane host selects between stereoisomers. Angew. Chem. Int. Ed..

[14] Forgan R.S., Sauvage J.P., Stoddart J.F. (2011). Chemical topology: Complex molecular knots, links, and entanglements. Chem. Rev..

[15] Dang L.L., Sun Z.B., Shan W.L., Lin Y.J., Li Z.H., Jin G.X. (2019). Coordination-driven self-assembly of a molecular figure-eight knot and other topologically complex architectures. Nat. Commun..

[16] Wang H., Qin J., Huang C., Han Y., Xu W., Hou H. (2016). Mono/bimetallic water-stable lanthanide coordination polymers as luminescent probes for detecting cations, anions and organic solvent molecules. Dalton Trans..

[17] Zhang H.N., Gao W.X., Lin Y.J., Jin G.X. (2019). Reversible structural transformation between a molecular solomon link and an unusual unsymmetrical trefoil knot. J. Am. Chem. Soc..

[18] Dang L.L., Li T.T., Cui Z., Sui D., Ma L.F., Jin G.X. (2021). Selective construction and stability studies of a molecular trefoil knot and Solomon link. Dalton Trans..

[19] Lu Y., Liu D., Lin Y.J., Li Z.H., Hahn F.E., Jin G.X. (2021). An “All-in-One” Synthetic Strategy for Linear Metalla[4]Catenanes. J. Am. Chem. Soc..

[20] Xue X., Wang H., Han Y., Hou H. (2018). Photoswitchable nonlinear optical properties of metal complexes. Dalton Trans..

[21] Gao X., Cui Z., Shen Y.R., Liu D., Lin Y.J., Jin G.X. (2021). Synthesis and Near-Infrared photothermal conversion of discrete supramolecular topologies featuring Half-Sandwich [Cp*Rh] units. J. Am. Chem. Soc..

[22] Gao W.X., Feng H.J., Lin Y.J., Jin G.X. (2019). Covalent Post-assembly modification triggers structural transformations of borromean rings. J. Am. Chem. Soc..

[23] Gao W.X., Feng H.J., Guo B.B., Lu Y., Jin G.X. (2020). Coordination-Directed Construction of Molecular Links. Chem. Rev..

[24] Cui Z., Lu Y., Gao X., Feng H.J., Jin G.X. (2020). Stereoselective synthesis of a topologically chiral solomon link. J. Am. Chem. Soc..

[25] Caprice K., Pupier M., Bauzá A., Frontera A., Cougnon F.B.L. (2019). Synchronized on/off switching of four binding sites for water in a molecular solomon link. Angew. Chem. Int. Ed..

[26] Crowley J.D., Goldup S.M., Lee A., Leigh D.A., McBurney R.T. (2009). Active metal template synthesis of rotaxanes, catenanes and molecular shuttles. Chem. Soc. Rev..

[27] Tamura Y., Takezawa H., Fujita M. (2020). A Double-Walled knotted cage for Guest-Adaptive molecular recognition. J. Am. Chem. Soc..

[28] Sun L.Y., Sinha N., Yan T., Wang Y.S., Tan T.T.Y., Yu L., Han Y.F., Hahn F.E. (2018). Template synthesis of Three-Dimensional hexakisimidazolium cages. Angew. Chem. Int. Ed..

[29] Gosselin A.J., Rowland C.A., Bloch E.D. (2020). Permanently microporous Metal–Organic polyhedra. Chem. Rev..

[30] Dang L.L., Li T.T., Zhang T.T., Zhao Y., Chen T., Gao X., Ma L.F., Jin G.X. (2022). Highly selective synthesis and near-infrared photothermal conversion of metalla-Borromean ring and [2]catenane assemblies. Chem. Sci..

[31] Qin J.H., Huang Y.D., Zhao Y., Yang X.G., Li F.F., Wang C., Ma L.F. (2019). Highly dense packing of chromophoric linkers achievable in a Pyrene-Based Metal–Organic framework for photoelectric response. Inorg. Chem..

[32] Yang X.G., Liu X.Y., Zhai Z.M., Qin J.H., Chang X.H., Han M.L., Li F.F., Ma L.F. (2020). π-Type halogen bonding enhanced long-last room temperature phosphorescence of Zn(II) coordination polymers for photoelectron response applications. Inorg. Chem. Front..

[33] Dang L.L., Li T.T., Zhao C.C., Zhang T.T., Ye X.Y., Sun X.T., Wang H.R., Ma L.F. (2022). Supramolecular Rh6 catalytic system promoting directed [4 + 4] cycloaddition reaction of anthracene under UV irradiation. J. Solid State Chem..

[34] Qin J.H., Xu P., Huang Y.D., Xiao L.Y., Lu W., Yang X.G., Ma L.F., Zang S.Q. (2021). High loading of Mn(II)-metalated porphyrin in MOF for photocatalytic CO_2_ reduction in gas−solid condition. Chem. Commun..

[35] Lu Y., Liu D., Cui Z., Lin Y.J., Jin G.X. (2021). Adaptive Self-Assembly and Induced-Fit interconversions between molecular borromean rings, russian dolls and Ring-in-Ring complexes. Chin. J. Chem..

[36] Dang L.L., Gao X., Lin Y.J., Jin G.X. (2020). s-Block metal ions induce structural transformations between figure-eight and double trefoil knots. Chem. Sci..

[37] Zhang H.N., Yu W.B., Lin Y.J., Jin G.X. (2021). Stimuli-Responsive topological transformation of a molecular borromean ring via controlled oxidation of thioether moieties. Angew. Chem. Int. Ed..

[38] Song Y.H., Singh N., Jung J., Kim H., Kim E.H., Cheong H.K., Kim Y., Chi K.W. (2016). Template-Free synthesis of a molecular solomon link by Two-Component Self-Assembly. Angew. Chem. Int. Ed..

[39] Sheldrick G.M. (1990). Phase annealing in SHELX-90: Direct methods for larger structures. Acta Crystallogr. Sect. A.

[40] Sheldrick G.M. (2008). A short history of SHELX. Acta Crystallogr. Sect. A.

[41] Li T.T., Dang L.L., Zhao C.C., Lv Z.Y., Yang X.G., Zhao Y., Zhang S.H. (2021). A self-sensitized Co (II)-MOF for efficient visible-light-driven hydrogen evolution without additional cocatalysts. J. Solid State Chem..

[42] Wang H.R., Yang X.G., Qin J.H., Ma L.F. (2021). Long-lived room temperature phosphorescence of organic–inorganic hybrid systems. Inorg. Chem. Front..

[43] Shan W.L., Gao W.X., Lin Y.J., Jin G.X. (2018). Light-initiated reversible conversion of macrocyclic endoperoxides derived from half-sandwich rhodium-based metallarectangles. Dalton Trans..

[44] Qin J.H., Zhang H., Sun P., Huang Y.D., Shen Q., Yang X.G., Ma L.F. (2020). Ionic liquid induced highly dense assembly of porphyrin in MOF nanosheets for photodynamic therapy. Dalton Trans..

[45] Yang X.G., Lu X.M., Zhai Z.M., Zhao Y., Liu X.Y., Ma L.F., Zang S.Q. (2019). Facile synthesis of micro-scale MOF host-guest with long-last phosphorescence and enhanced optoelectronic performance. Chem. Commun..

[46] Yang X.G., Zhai Z.M., Liu X.Y., Qin J.H., Li F.F., Ma L.F. (2020). Hexanuclear Zn(II) induced dense π-stacking in MOF featuring long-last room temperature phosphorescence. Inorg. Chem..

[47] Wang H., Meng W., Wu J., Ding J., Hou H., Fan Y. (2016). Crystalline central-metal transformation in metal-organic frameworks. Coord. Chem. Rev..

[48] Wang Y.F., Li S.H., Ma L.F., Geng J.L., Wang L.Y. (2015). Syntheses, crystal structures, and magnetic studies of two cobalt(II) coordination polymers based on concurrent ligand extension. Inorg. Chem. Commun..

[49] Chang X.H., Qin W.J., Zhang X.Y., Jin X., Yang X.G., Dou C.X., Ma L.F. (2021). Angle-Dependent Polarized Emission and Photoelectron Performance of Dye-Encapsulated Metal-Organic Framework. Inorg. Chem..

[50] Fu H.R., Yan L.B., Wu N.T., Ma L.F., Zang S.Q. (2018). Dual-emission MOF Édye sensor for ratiometric fluorescence recognition of RDX and detection of a broad class of nitro-compounds. J. Mater. Chem. A.

[51] Qin J.H., Qin W.J., Xiao Z., Yang J.K., Wang H.R., Yang X.G., Li D.S., Ma L.F. (2021). Efficient energy-transfer-induced high photoelectric conversion in a dye-encapsulated ionic pyrene-based metal–organic framework. Inorg. Chem..

[52] Dang L.L., Zhang T.T., Li T.T., Chen T., Zhao Y., Zhao C.C., Ma L.F. (2022). Stable Zinc-Based Metal-Organic Framework Photocatalyst for Effective Visible-Light-Driven Hydrogen Production. Molecules.

[53] Zhao Y., Yang X.G., Lu X.M., Yang C.D., Fan N.N., Yang Z.T., Wang L.Y., Ma L.F. (2019). {Zn_6_} Cluster Based Metal–Organic Framework with Enhanced Room-Temperature Phosphorescence and Optoelectronic Performances. Inorg. Chem..

[54] Chang X.H., Qin J.H., Han M.L., Ma L.F., Wang L.Y. (2014). Exploring the structural diversities and magnetic properties of copper(II) and manganese(II) complexes based on 5-methoxyisophthalate and flexible bis(imidazole) ligands. Crystengcomm..

[55] Wang Y.F., Tai J.H., Yan X.W., Zhao M.Y., Wang L.Y. (2018). Crystal structures and magnetic properties of two isomorphic frameworks based on 3-(1H-pyrazol-4-yl)-5-(pyridin-2-yl)-1, 2, 4-triazole and 1,2,4,5-benzenetetracarboxylic acid. Chin. J. Inorg. Chem..

[56] Wang Y.F., He C.J. (2018). Syntheses, Crystal Structures and Characterization of Two Coordination Polymers Based on Mixed Ligands. Chin. J. Struct. Chem..

[57] Chang X.H., Ling X.L., Lu X.M., Yang X.G., Li F.F., Guo Y.M. (2020). Near-infrared phosphorescence emission of three-fold interpenetrated MOF based on 1,4-bis(imidazole-1-ylmethyl)benzene: Syntheses, structure and photoelectron performance. J. Solid State Chem..

[58] Wu X.X., Fu H.R., Han M.L., Zhou Z., Ma L.F. (2017). Tetraphenylethylene Immobilized Metal−Organic Frameworks: Highly Sensitive Fluorescent Sensor for the Detection of Cr_2_O_7_^2−^ and Nitroaromatic Explosives. Cryst. Growth Des..

[59] Chang X.H., Yang X.G., Zhai Z.M., Chen J.Y., Li F.F. (2021). Synthesis, Structure and Highly Enhanced Phosphorescence of a Cadmium(II) Coordination Polymer Assembled with 1,4-Naphthalenedicarboxylic Acid and 2-Propylimidazole. Chin. J. Struc. Chem..

[60] Zhao Y., Wang L., Fan N.N., Han M.L., Yang G.P., Ma L.F. (2018). Porous Zn(II)-Based Metal–Organic Frameworks Decorated with Carboxylate Groups Exhibiting High Gas Adsorption and Separation of Organic Dyes. Cryst. Growth Des..

