# Supplementary material for: Size-Induced Highly Selective Synthesis of Organometallic Rectangular Macrocycles and Heterometallic Cage Based on Half-Sandwich Rhodium Building Block
Source: Molecules. 2022 Jun 10;27(12):3756. doi: 10.3390/molecules27123756 (PMC9230013; doi:10.3390/molecules27123756)
Supplement: Supplementary file 1 [file molecules-27-03756-s001.zip › molecules-1753699-supplementary.pdf]

Supplementary Materials for

Size-Induced Highly Selective Synthesis of Organometallic Rectangular Macrocycles and Heterometallic Cage Based on Half-Sandwich Rhodium Building Block

Li-Long Dang [1,2,*], Tian Chen [1,3], Ting-Ting Zhang [1], Ting-Ting Li [1], Jun-Liang Song [1], Ke-Jia Zhang [1] and Lu-Fang Ma [1,2]

1. Henan Province Function-Oriented Porous Materials Key Laboratory,

College of Chemistry and Chemical Engineering, Luoyang Normal University,

Luoyang 471934, China

2. Shanghai Key Laboratory of Molecular Catalysis and Innovative Materials,

Fudan University, Shanghai 200438, China

3. College of Chemistry, Zhengzhou University, Zhengzhou 450001, China

* Correspondence: danglilong8@163.com

A. Materials and Methods

All reagents and solvents were obtained commercially and used without further purification. The starting materials $[Cp^*RhCl(\mu\text{-}Cl)]_2$ (**E1**), $[Cp^*_2Rh_2(BiBzlm)](Cl)_2$ (**E2**) and $[Cp^*_2Rh_2(L^{Cu})]Cl_2$ (**E3**) were prepared according to literature methods. NMR spectra were recorded on Bruker AVANCE I 400 spectrometers at room temperature and referenced to the residual protonated solvent. Elemental analyses were performed on an Elementar Vario EL III analyzer. IR spectra of the solid samples (KBr tablets) in the range 400–4000 cm−1 were recorded on a Nicolet AVATAR-360IR spectrometer.

Single crystals of **1**, **2** and **3@2iPr$_2$O**, suitable for X-ray diffraction study were obtained at room temperature. X-ray intensity data were collected at 173 K on a CCD-Bruker SMART APEX system with Cu-Kα radiation (λ = 1.54178 Å). In these data, the disordered solvent molecules which could not be restrained properly were removed using the PLATON Squeeze routine. CCDC: 2173314 (**1**), 2173315 (**2**), 2173316 (**3@2iPr$_2$O**).

1. Preparation of metallarectangle 1

AgOTf (51.4 mg, 0.2 mmol) was added to a solution of $[Cp^*RhCl_2]_2$ (62.0 mg, 0.1 mmol) in CH_3OH (10 mL) at room temperature. The reaction mixture was stirred in the dark for 6 h and then filtered, followed by addition of **L1** (31.2 mg, 0.1 mmol). After stirring for another 12 h at room temperature, the reaction mixture was concentrated to a volume of 3 mL under reduced pressure. The product was recrystallized from a CH_3OH/ isopropyl ether mixture to afford yellow block-shaped crystals in 86 % yield. Elemental analysis calcd (%) for $C_{80}H_{84}Cl_4F_{12}N_{12}O_{12}Rh_4S_4$: C, 41.50; H, 3.66; N, 7.26; found: C, 41.54; H, 3.62; N, 7.21.^{1}H NMR (500 MHz, CD_3OD, ppm, with respect to Cp*Rh) δ = 8.20 (d, J = 5.6Hz, 8H, pyridyl-aH), 8.11 (d, J = 5.5 Hz, 8H, pyridyl-bH), 7.29 (s, 4H, phenyl-cH), 1.65 (s, 60H, Cp*-H). IR (KBr cm^{-1}): 3441, 2926, 1615, 1445, 1425, 1393, 1279, 1225, 1164, 1030, 837, 639, 605, 574.

2. Preparation of metallarectangle 2

AgOTf (51.4 mg, 0.2 mmol) was added to a solution of $[Cp^*_2Rh_2(BiBzlm)](Cl)_2$ (63.9 mg, 0.1 mmol) in CH_3OH (10 mL) at room temperature. The reaction mixture was allowed to stir under dark conditions for 8 h and then filtered, followed by addition of **L1** (31.2 mg, 0.1 mmol). After stirring for another 12 h at 298K, the final mixed solution was concentrated to a volume of about 3 mL under the condition of reduced pressure. Thus, the product was recrystallized from a CH_3OH/ isopropyl ether mixture to afford yellow block-shaped crystals in 83% yield. Anal. Calcd for $C_{110}H_{108}F_{12}N_{20}O_{14}Rh_4S_4$ (M = 2686.16): C, 48.90; H, 4.03; N, 10.37. Found: C, 48.96; H, 4.08; N, 10.31. ^{1}H NMR (500 MHz, CD_3CD, ppm, with respect to Cp*Rh): δ = 8.18-8.17 (m, 8H, BiBzIm-eH), δ = 7.64-7.62 (m, 8H, BiBzIm-fH), δ = 7.87 (d, J = 6.0Hz, 8H, pyridyl-aH), δ = 7.39

(d, J = 5.5 Hz, 8H, pyridyl-bH), δ = 7.08 (s, 4H, phenyl-cH), δ = 1.92 (s, 60H, Cp*-H). IR (KBr cm^{-1}): 3442, 2924, 1612, 1447, 1286, 1241, 1163, 1029, 831, 745, 635, 572, 514.

3. Preparation of heterometallic cage 3@2iPr$_2$O

AgOTf (51.4 mg, 0.2 mmol) was added to a solution of [Cp*$_2$Rh$_2$(L^{Cu})]Cl$_2$ (81.1 mg, 0.1 mmol) in CH_3OH (10 mL) at room temperature. The reaction mixture was stirred in the dark for 6 h and then filtered, followed by addition of **L1** (15.6 mg, 0.05 mmol). After stirring for another 12 h at room temperature, the reaction mixture was concentrated to a volume of 3 mL under reduced pressure. The product was recrystallized from a CH_3OH/ isopropyl ether mixture to afford green crystals in 82% yield. Anal. Calcd for $C_{176}H_{192}Cu_4F_{24}N_{20}O_{54}Rh_8S_8$ (M = 5241.40): C, 48.90; H, 4.03; N, 10.37. Found: C, 48.96; H, 4.08; N, 10.31. IR (KBr cm^{-1}): 3443, 2924, 1623, 1422, 1279, 1159, 1026, 869, 759, 639, 574, 521.

B. Supplementary Table

Table S1. Crystallographic data for **1**, **2** and **3**.

	**1**	**2**	**3**
formula	$C_{80}H_{84}Cl_4F_{12}N_{12}O_{12}Rh_4S_4$	$C_{110}H_{108}F_{12}N_{20}O_{14}Rh_4S_4$	$C_{176}H_{192}Cu_4F_{24}N_{20}O_{54}Rh_8S_8$
Mr	2315.27	2702.04	5241.40
crystal system	monoclinic	Triclinic	Triclinic
space group	*P*21/*c*	*P*-1	*P*-1
a [Å]	23.0473(4)	12.3037(10)	16.5897(16)
b [Å]	18.2050(3)	13.1305(10)	20.863(2)
c [Å]	29.1160(5)	21.4434(16)	21.156(2)
α [º]	90	79.849(4)	69.566(6)
β [º]	104.0540(10)	74.436(4)	78.390(7)
γ [º]	90	64.969(3)	66.851(6)
V [$Å^3$]	11850.7(4)	3016.0(4)	6291.7(11)
T [K]	173(2)	173(2)	173(2)
Z	4	1	1
ρ_{calcd} [g cm^{-3}]	1.298	1.488	1.383
μ [mm^{-1}]	6.514	3.848	5.846
F(000)	4656.0	1372.0	2640.0
independent	0.0801	0.0736	0.1759
data/restraints/para	23290/3280/116	11380/2302/806	23875/2807/134
R_1/wR_2 [I>2$\sigma(I)$][a]	0.0951/0.2151	0.1190/0.2554	0.1518/0.3407
R_1/wR_2 (all data)[a]	0.1279/ 0.2355	0.1478/0.2684	0.2280/0.3778
goodness-of-fit	1.108	1.170	1.074
largest residuals [e Å-	3.47/-1.90	4.51/-1.61	3.44/-3.06

[a] $R_1 = \Sigma(||F_o| - |F_c||)/\Sigma|F_o|$; [b] $wR_2 = [\Sigma w(|F_o|^2 - |F_c|^2)^2/\Sigma w(F_o^2)^2]^{1/2}$
